# Mining biosynthetic gene clusters in *Virgibacillus* genomes

**DOI:** 10.1186/s12864-019-6065-7

**Published:** 2019-09-03

**Authors:** Ghofran Othoum, Salim Bougouffa, Ameerah Bokhari, Feras F. Lafi, Takashi Gojobori, Heribert Hirt, Ivan Mijakovic, Vladimir B. Bajic, Magbubah Essack

**Affiliations:** 1 Computational Bioscience Research Center (CBRC), Computer, Electrical and Mathematical Sciences and Engineering (CEMSE) Division, King Abdullah University of Science and Technology (KAUST), Thuwal, 23955-6900 Kingdom of Saudi Arabia; 2 McDonnell Genome Institute, Washington University School of Medicine, St. Louis, MO 63110 USA; 3 Biological and Environmental Sciences and Engineering (BESE) Division, King Abdullah University of Science and Technology (KAUST), Thuwal, 23955-6900 Kingdom of Saudi Arabia; 4 College of Natural and Health Sciences, Zayed University, Abu-Dhabi, 144534 United Arab Emirates; 5 Division of Systems & Synthetic Biology, Department of Biology and Biological Engineering, Chalmers University of Technology, Kemivägen 10, 41296 Gothenburg, Sweden; 6 Novo Nordisk Foundation Center for Biosustainability, Technical University of Denmark, 2800 Lyngby, Denmark

**Keywords:** *Virgibacillus*, Antimicrobial, Biosynthetic gene clusters, Genome-mining, Nonribosomal peptides, Polyketides, Bacteriocins, Lanthipeptides, Bioinformatics

## Abstract

**Background:**

Biosynthetic gene clusters produce a wide range of metabolites with activities that are of interest to the pharmaceutical industry. Specific interest is shown towards those metabolites that exhibit antimicrobial activities against multidrug-resistant bacteria that have become a global health threat. Genera of the phylum Firmicutes are frequently identified as sources of such metabolites, but the biosynthetic potential of its *Virgibacillus* genus is not known. Here, we used comparative genomic analysis to determine whether *Virgibacillus* strains isolated from the Red Sea mangrove mud in Rabigh Harbor Lagoon, Saudi Arabia, may be an attractive source of such novel antimicrobial agents.

**Results:**

A comparative genomics analysis based on *Virgibacillus dokdonensis* Bac330, *Virgibacillus sp.* Bac332 and *Virgibacillus halodenitrificans* Bac324 (isolated from the Red Sea) and six other previously reported *Virgibacillus* strains was performed. Orthology analysis was used to determine the core genomes as well as the accessory genome of the nine *Virgibacillus* strains. The analysis shows that the Red Sea strain *Virgibacillus sp.* Bac332 has the highest number of unique genes and genomic islands compared to other genomes included in this study. Focusing on biosynthetic gene clusters, we show how marine isolates, including those from the Red Sea, are more enriched with nonribosomal peptides compared to the other *Virgibacillus* species. We also found that most nonribosomal peptide synthases identified in the *Virgibacillus* strains are part of genomic regions that are potentially horizontally transferred.

**Conclusions:**

The Red Sea *Virgibacillus* strains have a large number of biosynthetic genes in clusters that are not assigned to known products, indicating significant potential for the discovery of novel bioactive compounds. Also, having more modular synthetase units suggests that these strains are good candidates for experimental characterization of previously identified bioactive compounds as well. Future efforts will be directed towards establishing the properties of the potentially novel compounds encoded by the Red Sea specific *trans*-AT PKS/NRPS cluster and the type III PKS/NRPS cluster.

**Electronic supplementary material:**

The online version of this article (10.1186/s12864-019-6065-7) contains supplementary material, which is available to authorized users.

## Background

Biosynthetic gene clusters (BGCs), made up of multi-enzymatic, multi-domain megasynthases, are often of interest in genome-mining. For instance, the most well studied modular clusters, nonribosomal peptide synthetases (NRPSs) and Polyketide Synthases (PKSs) are often associated with the synthesis of antitumor, antimicrobial, antifungal, and immunosuppressive products [1, 2]. Another BGC class is the ribosomally synthesized and posttranslationally modified peptides (RiPPs) [3] which include the extensively studied bacteriocins and lanthipeptides [3], both of which have known products with a wide spectrum of antimicrobial activity. In fact, lanthipeptides were initially termed lantibiotics because the first identified clusters exhibited antibiotic activity. But, as more and more clusters without antibiotic activity were discovered, the more generic term lanthipeptide came into use. A well-described example of anon-antibiotic lanthipeptide is SapB identified in *Streptomyces coelicolor* [4]. Nonetheless, other RiPPs also exhibit antimicrobial activity including thiopeptides, bottromycins, lipolanthines, etc.

Because of this knowledge and the increase in available sequenced genomes, methods have been developed to allow mining of sequenced genomes for these BGCs [5–11]. However, there are no studies that provide insights into genomic features in strains belonging to the *Virgibacillus* genus of the Firmicutes phylum. This is surprising owing to the fact that: 1/ the rod-shaped, endospore-forming species in this genus are both gram-stain variable and gram-stain positive, 2/ these strains exhibit an ability to adapt to diverse environments such as marine sediment [12], faeces [13] and fermented food [14, 15], 3/ these strains exhibit enzymatic and antimicrobial potential of interest to industry [15–20], and 4/ there is a large number of sequenced *Virgibacillus* genomes (31 at the time of conducting our study, with six complete genomes assembled).

We previously reported [20] that *V. dokdonensis* Bac330 and *Virgibacillus sp.* Bac332 exhibited antimicrobial activity against both *Staphylococcus aureus* (ATCC25923) and *Pseudomonas syringae* (dc3000), respectively, while *V. halodenitrificans* Bac324 displayed no such activity. These strains were isolated from Red Sea locations which are shown through metagenomic analysis [21] to harbor a rich repertoire of NRPS and PKS sequences. The nature of the environment (high salinity and temperature) can be a contributing factor to the horizontal transfer of mobile genetic elements contributing to strengthening the biosynthetic potential of its microbiome. Here, we identify features in these three *Virgibacillus* strains isolated from the Red Sea, which provide insights into strains’ biosynthetic potential encoded by their genomes. Based on the comparison with other strains from the same genus, we show specific genetic characteristics unique to the marine *Virgibacillus* strains, including Red Sea strains.

## Results and discussion

### Features of the genomes of the Red Sea *Virgibacillus* strains

**Fig. 1:**
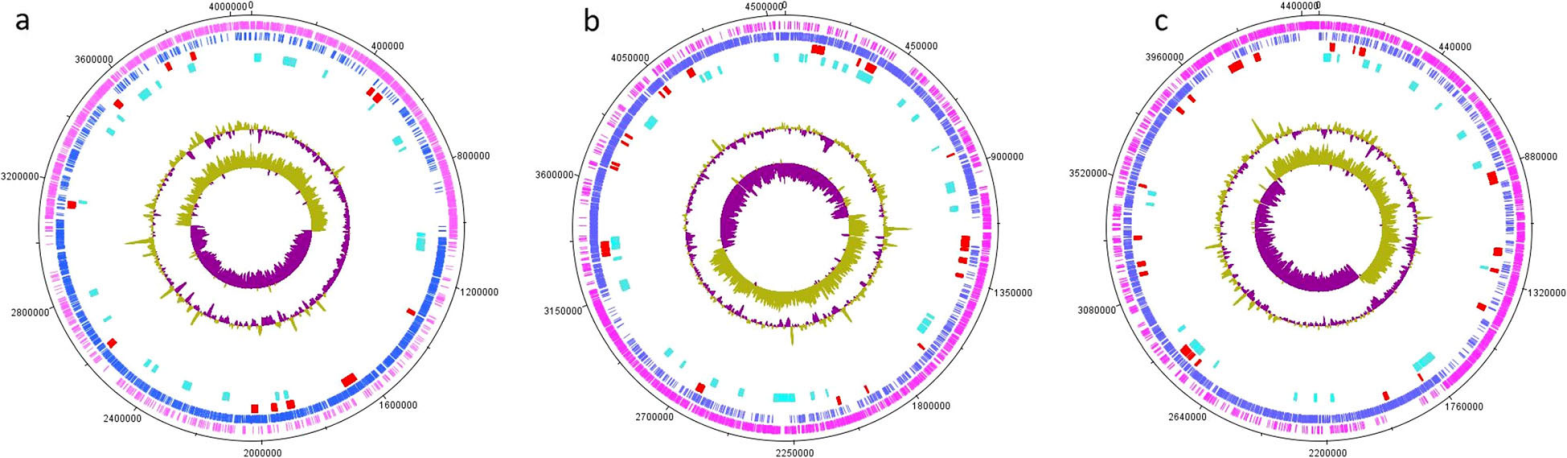
Circular plots of (**a**) Bac330 and (**b**) Bac324 and (**c**) Bac332 genomes, showing the overlap of biosynthetic genes and genomic islands. The tracks show the following features starting from the outermost track; 1st track (pink): genes on the positive strand; 2nd track (blue): genes on the negative strand; 3rd track (yellow): biosynthetic gene clusters; 4th track (red): horizontally transferred genes; 5th track (cyan): genes in prophage regions; 6th track: GC-plot where purple and green correspond to below and above average GC content, respectively; 7th track: GC-skew where purple and green correspond to below and above average GC-skew, respectively

Raw sequences of the three *Virgibacillus* genomes- *V. dokdonensis* Bac330 (CP033048), *Virgibacillus sp.* Bac332 (CP033046-CP033047), and *V. halodenitrificans* Bac324 (CP033049- CP033050)- showed that on average there are 133,749.6667 reads per genome (141,676, 148,002, 111,571 reads for *V. dokdonensis* Bac330, *Virgibacillus sp.* Bac332, and *V. halodenitrificans* Bac324, respectively). The read mean length was 7952 bp (253x genome coverage) for *V. dokdonensis* Bac330*,* 8939 bp (290x genome coverage) for *Virgibacillus sp.* Bac332 and 11,324 bp (311x genome coverage) for *V. halodenitrificans* Bac324. Assemblies of the reads showed that two of the strains have plasmids (*Virgibacillus sp.* Bac332 and *V. halodenitrificans* Bac324), while *V. dokdonensis* Bac330 only has one circular chromosome without plasmid. *V. dokdonensis* Bac330’s circular chromosome was 4,456,326 bp in length with 4163 predicted open reading frames (ORFs) (52.3% on the forward strand, and 47.7% on the reverse complement strand). *V. halodenitrificans* Bac324 circular chromosome was 4,063,118 bp in length with 4284 predicted ORFs (46.9% on the forward strand and 53.1% on the reverse complement one) and its plasmid was 312,876 bp in length. *Virgibacillus sp.* Bac332 circular chromosome was 4,561,556 bp in length with 4424 predicted ORFs (46.1% on the forward strand and 53.9% on the reverse complement one) and its plasmid was 65,691 bp in length. The total number of tRNAs was almost the same in the three genomes (63,64,64 tRNAs), while the number of rRNAs was 18 for both *Virgibacillus sp.* Bac332 and *V. dokdonensis* Bac330 but was 24 for *V. halodenitrificans* Bac324 (Table 1, Fig. 1).

**Table 1:** Summary of the genomes and annotation of nine Virgibacillus strains

Strain	Isolation site	Number of ORFs	Genome size (Mb)	GC%	Number of scaffolds	Genomic Islands %	# of rRNAs	# of tRNAs	# of unique genes
*Virgibacillus sp.* SK37 (Genbank accession number: CP007161)	Fish sauce mash	3795	3.84	37.59	4	4.9	21	53	504
*Virgibacillus halodenitrificans* PDB-F2 (Genbank accession number: CP017962)	Solid waste landfill	3820	3.92	37.43	2	2.8	24	62	255
*Virgibacillus phasianinus* LM2416 (Genbank accession number: CP022315)	Host *Lophura swinhoei*	3966	4.07	39.50	1	4.1	21	64	648
*Virgibacillus necropolis* LMG 19488 (Genbank accession number: CP022437)	Mural paintings	4135	4.34	37.30	1	2.2	22	62	761
*Virgibacillus dok0064onensis* 21D (Genbank accession number: CP018622)	Marine	3939	4.26	36.60	1	5.4	18	63	435
*Virgibacillus* sp. 6R (Genbank accession number: CP017762)	Coal bed	4313	4.75	37.30	1	5.9	18	65	927
*Virgibacillus halodenitrificans* Bac324 (Genbank accession number: CP033049)	Mangrove mud/Rabigh Harbour Lagoon	4284	4.06	37.2	2	5.19	24	62	665
*Virgibacillus sp*. Bac332 (Genbank accession number: CP033046)	mangrove mud	4424	4.56	36.7	2	9.32	18	63	958
*Virgibacillus dokdonensis* Bac330 (Genbank accession number: CP033048)	Mangrove mud	4163	4.46	36.8	1	6.9	18	64	619

**Fig. 2:**
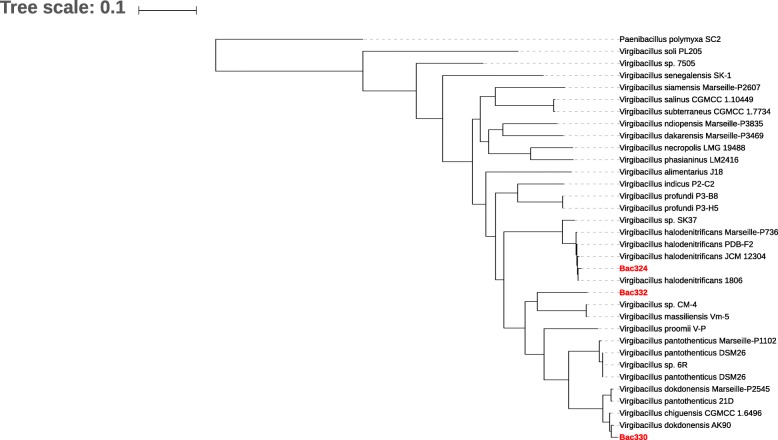
Maximum-likelihood phylogenetic tree of 32 genomes constructed using 606 single-copy genes. *Paenibacillus polymyxa* was used as the outgroup. The tree shows the phylogenetic proximity of the Red Sea *Virgibacillus* strains in the *Virgibacillus* group

For phylogenetic placement of the three Red Sea strains, a phylogenetic tree was generated using 606 single-copy genes (Fig. 2). We included a total of 31 complete and incomplete *Virgibacillus* genomes for a higher resolution in the placement. We also used *Paenibacillus polymyxa* as the outgroup.

The resulting tree (Fig. 2) shows the phylogenetic proximity Bac330, Bac324, and Bac332 to *Virgibacillus* strains as indicated by the 16S rRNA analysis reported in a previous study [20].

To identify the unique functional groups in the three complete *Virgibacillus* genomes from the Red Sea, we investigated the accessory genome of these isolates and searched for orthologous families that have genes exclusively present in any genome of the three Red Sea *Virgibacillus* strains and are consistently absent from the six complete publicly available *Virgibacillus* genomes. The analysis showed that amongst the 11,135 gene families that constitute the pan-genome of the analyzed strains, there are 958 unique genes in *Virgibacillus sp.* Bac332. There are 665 unique genes in *V. halodenitrificans* Bac324 and 619 in *V. dokdonensis* Bac330 (See Table 1). The number of unique genes in the non-Red Sea *Virgibacillus* strains is 255 in *V. halodenitrificans* PDB-F2, 761 in *V. necropolis* LMG 19488, 435 in *V. dokdonensis* 21D, 504 in *Virgibacillus sp.* sk37, 927 in *Virgibacillus sp.* 6R and 648 in *V. phasianinus* LM2416. Thus, *Virgibacillus sp.* Bac332 has the highest number of unique genes among the nine genomes.

**Fig. 3:**
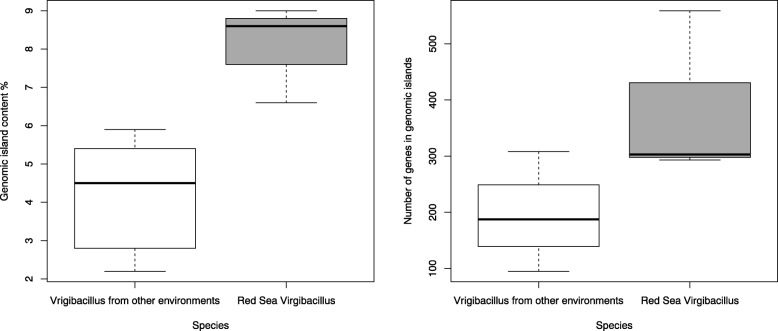
Boxplot of genomic island content and number of genes in genomic islands falling in the public *Virgibacillus* strains (white) and Red Sea *Virgibacillus* (grey) using both the size of the DNA regions in which predicted GIs fall as well as the number of predicted genes

Since horizontal gene transfer is considered central to microbes’ ability to adapt to an ecological niche, we also predicted the genomic islands (GIs) in the analyzed genomes to increase our understanding as to how the three *Virgibacillus* strains have potentially acquired genomic elements adding to their biosynthetic capacity. Interestingly, the chromosomal sequence of *Virgibacillus sp.* Bac332 has the highest number of GIs with a total of 434,257 bp of DNA (21 GIs harboring 559 genes), equivalent to 9% of the genome size. GI prediction also identified 210,858 bp of the chromosomal sequence in the genome of *V. halodenitrificans* Bac324 (13 GIs harboring 293 genes) and 309,859 bp in *V. dokdonensis* Bac330 (19 GIs harboring 303 genes) amounting to 5.19 and 6.9% of the genomes, respectively. On average, there is 178,642 bp of DNA sequence in GI predicted regions in any of the publicly available chromosomal sequences of the six non-Red Sea *Virgibacillus* strains, where *V. necropolis* LMG 19488 has the lowest number of GIs (6 GIs) extending over 96,572 bp of DNA and harboring 95 genes. On the other hand, the genomes of the Red Sea isolates have on average 318,325 bp of GI sequence per genome (Fig. 3).

These analyses show that despite the large size of the core genome (1654 ORFs accounting for 40% of the average number of ORFs), one Red Sea strain has a large number of unique genes that are not orthologous to any other gene in the studied *Virgibacillus* genomes, at least using the imposed identity threshold (see the Materials and Methods). Moreover, the strikingly high GI content in the genomes of *Virgibacillus sp.* Bac332 and *V. dokdonensis* Bac330, along with the high number of biosynthetic genes and unique genes, collectively hint toward the presence of unique functional genomic features in marine *Virgibacillus* strains compared to other genomes used in this study. Although we could not collectively discriminate strains based on environment type only, we have shown increased uniqueness in the genomic features of one of the Red Sea strains, *Virgibacillus sp.* Bac332.

### Exploring the biosynthetic potential of the Red Sea *Virgibacillus* strains

To identify unique biosynthetic elements in the Red Sea strains that might indicate specific functions as a result of environmental adaptation, we had to confirm that the biosynthetic features identified in marine isolates, including ones from the Red Sea, are not present in closely related species. To do so, we included the six complete *Virgibacillus* species along with the three Red Sea strains for the evaluation (Table 1).

**Fig. 4:**
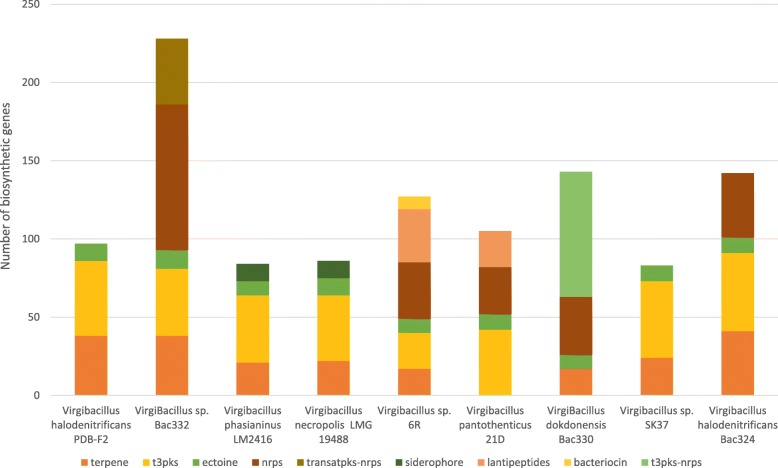
Distribution of genes in biosynthetic gene clusters in nine *Virgibacillus* genomes. Strains are color-coded as per the legend. The distribution clearly shows that genomes with the highest number of genes in BGCs are in the *Virgibacillus* Red Sea isolates

On average, each of the analyzed genomes comprises 24 putative biosynthetic gene clusters (BGCs) that were predicted by antiSMASH or *ClusterFinder*. The clusters predicted to fall in known classes of BGCs are mainly encoding for proteins related to the following biosynthetic classes: terpene, type III PKS, ectoine, NRPS, trans-acyltransferase PKS/NRPS, siderophore, lanthipeptide, bacteriocin, and type III PKS/NRPS (Fig. 4). Of interest to this study are two classes of secondary metabolites known for their use in different applications of pharmaceutical and industrial interest: 1/ modular clusters encompassing mainly NRPSs and modular PKS, and 2/ ribosomally synthesized peptides, namely bacteriocins and lanthipeptides.

#### Gene cluster families in Virgibacillus strains

A total of 215 BGCs (173 putative clusters and 42 in known BGC classes) with 3631 genes were classified into 35 groups (also referred to as gene cluster families GCFs) using scoring similarity networks as implemented in BiG-SCAPE [22] .

Interestingly, only three gene cluster families were assigned to clusters that produce known products or have a similar pathway using threshold similarity of 60% (these include paeninodin, locillomycin, and ectoine). Most notably, there is at least one terpene, type III PKS, and ectoine cluster in all of the genomes. Only one bacteriocin encoding BGC is identified in *Virgibacillus sp.* 6R, while lipopeptides were only identified in *Virgibacillus sp.* 6R and *V. dokdonensis* 21D. Siderophores were identified in *V. phasianinus* LM2416 and *V. necropolis* LMG 19488 only. The diversity of the distribution of BGC types across the *Virgibacillus* genomes, in spite of phylogenetic proximity, is an indication of the acquisition of genomic elements that enable biosynthetic routes of various products at different isolation sites. To investigate the emergence of environment-specific clusters, we looked at gene cluster families that are not shared by strains of the same species but shared between strains from different phylogenetic groups in the tree. In total, out of 35 gene cluster families, 10 are shared between at least one of the three Red Sea isolates and *V. dokdonensis* strain 21D; the only other marine isolate in the analysis (Additional file 1: Figure S1). Specifically, 6 of these clusters are exclusively shared between *V. dokdonensis* strain 21D and *V. dokdonensis* Bac330 or *Virgibacillus sp.* Bac332. Interestingly, other strains that have repeated patterns of shared clusters are either strains of the same species or strains falling in the same phylogenetic group. Additionally, Bac332 shares one cluster with *V. halodenitrificans* Bac324 and *V. halodenitrificans* PDB-F2, despite falling in different groups. Unfortunately, since none of these clusters have assigned products, we could not elaborate on the putative functions of these clusters.

#### Modular clusters and ribosomally synthesized and posttranslationally modified peptides (RiPPs)

In order to identify any exclusive modular clusters in the genomes of the marine isolates, we categorized the clusters based on gene homology and product type and assessed their distributions in the genomes. We found that out of a total of eight NRPSs and modular PKSs collectively identified in the nine *Virgibacillus* genomes, six are in the Red Sea *Virgibacillus* strains (two in *V. dokdonensis* Bac330, three in *Virgibacillus sp.* Bac332 and one in *V. halodenitrificans* Bac324) (Additional file 2: Table S1). The other two modular clusters are in *Virgibacillus sp.* 6R and in *V. dokdonensis* 21D. Only one of the clusters maps to known BGCs using a 60% similarity threshold (a hybrid *trans*-AT PKS/NRPS cluster in Bac332 with 64% similarity to Locillomycin). One NRPS cluster shares a number of structural features in four strains (Bac330, Bac324, 6R, and 21D), albeit with some variations hindering their assignment as the same cluster. Specifically, Bac324 and 6R have clusters in the same GCF with the highest similarity, while the cluster in 6R is the most variant with a single KS domain, and a cis-acting AT domain. The clusters in Bac324 and 6R have a combination of shared condensation (c) domains, adenylation (A) domains, as well as acylotransferase (AT) domains (that are putatively acting in *trans* due to their organization in the cluster as standalone domains). The presence of PKS domains such as ketosynthase (KS), ketoreductase (KR) in Bac330, 6R and 21D indicates that these could be a hybrid PKS/NRPS clusters. It is noteworthy that none of the hybrid PKS/NRPS clusters or NRPS ones in *Virgibacillus sp.* Bac332 share the same modular structure as other modular clusters in other *Virgibacillus* strains.

**Fig. 5:**
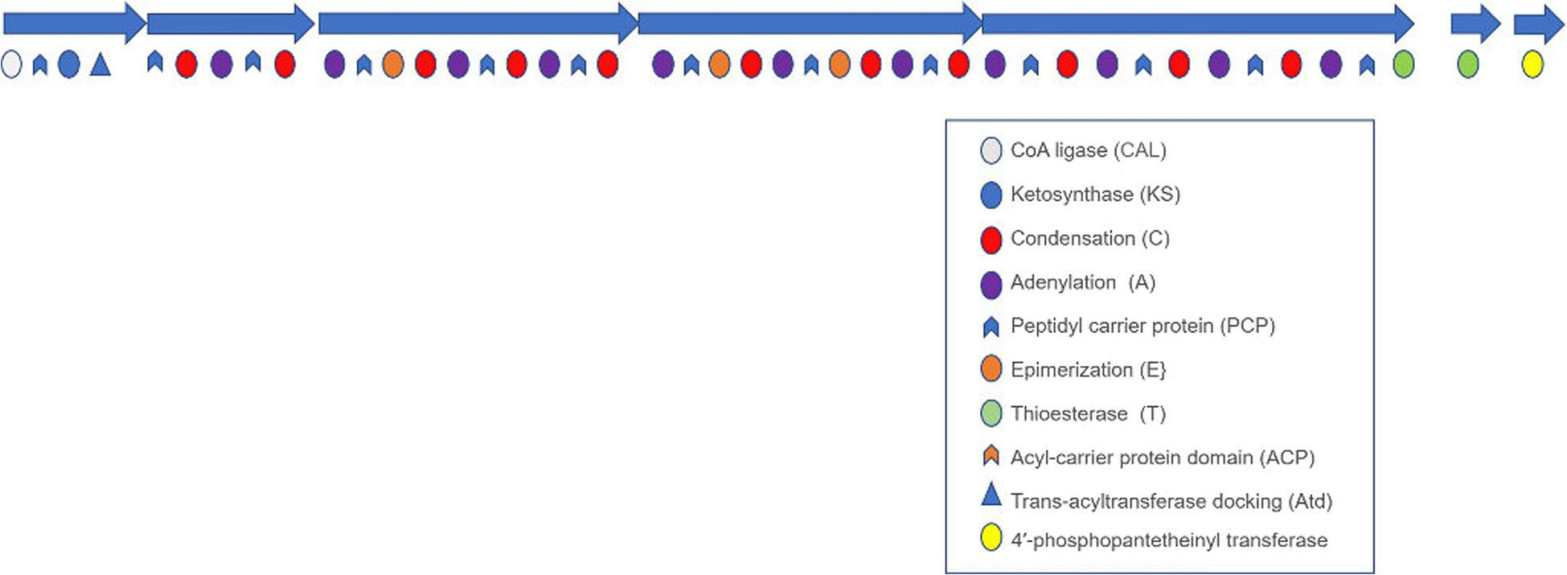
Structure of the hybrid PKS/NRPS cluster present in Bac332. Biosynthetic genes are identified with blue arrows. Domains abbreviations and color codes are shown in the legend

One uniquely structured cluster that has both PKS and NRPS domains is identified in the Red Sea *Virgibacillus sp.* Bac332. The cluster is characterized by the presence of four NRPS synthases composed of Adenylation (A), condensation (C) Epimerization (E), peptidyl carrier protein (PCP) domains and a thioesterase (T) domain. It also has one modular PKS synthase with a ketosynthase (KS) domain, a CoA ligase (CAL) domain and a *trans*-AT binding site (Fig. 5). There are also two single-domain peptides in the cluster: one with a 4′-phosphopantetheinyl transferase and one with another thioesterase. The cluster has a 64% similarity to the BGC encoding locillomycin. This product was previously reported in *Bacillus subtilis* 916 to be encoded by an unusual synthase that does not follow the collinearity rule of assembly-line clusters [23]. Despite the high similarity, we noted that the cluster in *Virgibacillus sp.* Bac332 has five additional NRPS modules, making it larger than the one in *B. subtilis* 916 (86 Kb in *Virgibacillus sp.* Bac332 and 38 Kb in *Bacillus subtilis* 916). We also noted that out of the five mega-synthases that make up the *trans*-AT PKS/NRPS cluster, four mapped to all of the known modular proteins in the locillomycin BGC (*LocA, LocB, LocC, and LocD*). The fact that the fifth 12,990 bp mega-synthase does not have an ortholog in the locillomycin cluster indicates putative novelty of the synthesized product.

There is also a large, uniquely-structured, hybrid Type III PKS/NRPS cluster in *V. dokdonensis* Bac330, and *Virgibacillus dokdonensis 21D*. The cluster is composed of 81 genes extending over 91 Kb of DNA. Specifically, there are three modular NRPS genes and three single-domain proteins as part of the type III PKS component of the cluster. This component has a 14% similarity to the cereulide BGC. However, none of the genes overlapping to the cereulide cluster are modular core NRPS ones (the genes mapping to the cereulide cluster are genes with an alpha/beta hydrolase domain and a gene encoding an ABC-2 type transport system binding protein). There are two bacteriocin and two class II lanthipeptide clusters in the analyzed genomes but none were identified in the Red Sea *Virgibacillus* species). The bacteriocin cluster has a glutamine synthetase, a *merR* family transcriptional regulator glutamine synthetase repressor, and one bacteriocin biosynthetic gene.

Taken together, when estimating the biosynthetic potential of these strains, we find that only a few of the identified clusters in the analyzed strains could be assigned to known products despite a total of 215 BGCs identified in the considered *Virgibacillus* genomes. Specifically, ~ 79% of the clusters per genome are putative and do not fall in known classes of BGCs. This is an indication that the biosynthetic potential of *Virgibacillus* species should be explored in much more details; there is an increasing number of publicly available genomes. Nonetheless, our results suggest that *Virgibacillus sp.* Bac332 has the highest number of modular genes falling in two NRPS clusters and a hybrid trans-AT PKS/NRPS cluster. Moreover, no bacteriocins or lanthipeptides were identified in the Red Sea isolates. The prevalence of modular clusters and low frequency of RiPPs in the Red Sea isolates could be part of an array of phenotypes required to adapt to the specific ecological conditions of the Red Sea. Moreover, the specific locations from which these strains were collected are in the Rabigh Harbor Lagoon, a substantial part of which has been converted into a harbor serving the Petro Rabigh petrochemical and refining complex and is therefore expected to be contaminated.

We specifically utilized the antiSMASH ClusterBlast module, as it allows us to assess the homology of the genes in the cluster to all genomes deposited in NCBI, either complete or draft. In Additional file 2: Table S1, we report the species with the top hits for these clusters. The fact that only a few of the top hits for the clusters in the Red Sea strains are *Virgibacillus* species supports our conclusions about the potential uniqueness of these clusters within the *Virgibacillus* genus. That is, most of the NRPS clusters (four out of six clusters) in the Red Sea strains have no match to any of the *Virgibacillus* genomes (complete or draft), but rather match some other distant *Bacillus* genomes, most of which are from saline environments. Moreover, one of the clusters in BAC332 had only 7% percent of its genes similar to a cluster in *Bacillus nakamurai* strain NRRL B-41091, indicating indeed that this is a unique cluster. Other clusters in the Red Sea strain have more than 60% identity to clusters in *Bacillus megaterium* MSP20.1 and *Tumebacillus* sp. AR23208. Modular clusters from the remaining *Virgibacillus* genomes (6R and 21D) were similar to clusters in *Marininema mesophilum* strain DSM 45610 and *Virgibacillus dokdonensis* strain Marseille-P2545, respectively. It is fair to summarize from the above analysis that at least one cluster in Bac332, is acquired by the strain as part of an environmental adaptation mechanism. This conclusion does not negate or conflict with the fact that other marine species from other environments might acquire similarly-structured clusters.

To further support the notion that Red Sea specific genes may be truly unique, we further performed homology analysis of modular genes (i.e., genes with NRPS or PKS domains) in the 6 clusters against MarCat from the MAR databases [24] (with Evalue:<1e-5; percent identity> 35%; and bitscore > 50, see Additional file 3: Table S2). Almost all of the modular genes have a hit greater than 30%, albeit one cluster often has different hits in different metagenome samples. This cannot be interpreted as an incomplete cluster as we can potentially attribute it to the incomplete nature of sequenced metagenomes. However, since overall, there is at least one significant hit in one metagenome per cluster, it is fair to attribute the uniqueness of the biosynthetic genes in the Red Sea genomes to marine environments in general.

### Biosynthetic gene clusters in genomic islands

The majority of the NRPS clusters in the Red Sea *Virgibacillus* genomes were found to fall in predicted GIs. Specifically, the NRPS identified in Bac332 is falling in a GI extending from 3,307,119 to 3,365,396 bp; while the NRPS cluster identified in Bac324 falls within the plasmid sequence identified in that genome. The NRPS cluster in *V. dokdonensis* Bac330 was found to overlap with a GI extending from 2,774,299 to 2,829,247 bp.

The fact that the majority of the identified NRPS clusters in the Red Sea isolates are part of GI regions motivated further investigation of the GIs in other *Virgibacillus* strains that have NRPS genes. Interestingly, we identified that the NRPS clusters in *Virgibacillus sp.* 6R (isolated from a Coal Bed) and *Virgibacillus pantothenticus* 21D (isolated from a marine environment) indeed are part of GIs that cover the region extending from1,078,422 to 1,129,3882 bp and from 2,579,235 to 2,619,409 bp, respectively. It is also noted that in most of the BGCs, there seems to be an overlap between the regions surrounding predicted BGC and GIs, which might indicate the acquisition of specialized genes necessary for the cluster, most of which are either transport elements or uncharacterized genes.

## Conclusions

Despite the availability of a total of 30 publicly available *Virgibacillus* genomes (at the time of the study), the biosynthetic potential of these strains has not been sufficiently explored. Species from marine environments, such as the Red Sea [25], were shown to harbor a number of promising clusters for the biosynthesis of modular antimicrobial clusters with unique structural properties. Here, we are analyzing *Virgibacillus* strains isolated from the Red Sea, along with publicly available *Virgibacillus* genomes, for their biosynthetic capabilities, leveraging the availability of complete genomes of the Red Sea isolates. To do so, we first computed the core and pangenome of nine *Virgibacillus* strains including the Red Sea ones. Our analysis shows that most of modular NRPS clusters in the analyzed genomes of *Virgibacillus* strains are part of horizontally transferred genomic regions in genomic islands. We also show that two of the Red Sea isolates (*V. dokdonensis* Bac330 and *Virgibacillus sp.* Bac332) collectively have more modular genes compared to all the analyzed genomes, indicating the possibility of the emergence of specific biosynthetic genomic elements in the genomes of these isolates in response to environmental selection in unique marine environments. Future efforts will be directed towards unveiling the biotechnological advantages of these isolates and in this process establishing the properties of the compounds encoded by the Red Sea specific *trans*-AT PKS/NRPS cluster and other NRPS clusters in Bac332. This would serve to highlight the novelty of the bioactive compounds, in terms of both function and structure, compared to other known compounds.

## Methods

### DNA extraction, sequencing, assembly, and annotation

A detailed description of sampling, isolation, and purification of the strains is available in [20]. Strains Bac330, Bac324 and Bac332 were isolated from mangrove mud samples collected from the Rabigh Harbor Lagoon in Saudi Arabia (39°0′35.762′′E, 22°45′5.582′′ N). Genome sequencing was performed at the Core Laboratory sequencing facility at KAUST using the PacBio RS II sequencing platform (Pacific Biosciences, USA) and assembled using PacBio’s SMRT Analysis pipeline v2.3.0. using default parameters. Prodigal [26] was used as the gene prediction method and genome annotation was completed using the Automatic Annotation of Microbial Genomes pipeline (AAMG) [27] with default parameters. A detailed description of sequencing, assembly, and annotation are available in [25].

### Comparative analysis of genomic features, genomic islands and biosynthetic genes

The core and pan-genomes were computed using GET_HOMOLOGUES v1.3 with the MCL option (−t 0 for core genome and -t all for pan-genome). We used a similarity threshold of 70%, alignment length coverage of 75% and e value of 1e-6. The number of core, shared and unique genes were visualized in an Upset fig. [28] as implemented in Intervene [29]. Prediction of GIs was completed using IslandViewer v4 [30, 31]. Circular visualization of the genomes and annotated features were plotted using DNAPlotter [32]. The phylogenetic tree was constructed using OrthoFinder v2.2.1 [33] utilizing gene trees for each orthogroup, with default settings, and visualized in iTOL [34]. Biosynthetic gene clusters were predicted using antiSMASH v3.0 [35] and cluster genes were mapped back to the MIBiG database in order to assign products to known clusters, with manual inspection of the focality of these alignments to core biosynthetic genes, as opposed to other accessory ones. Additionally, the *ClusterFinder* algorithm was also used to identify putatively novel clusters [36]*,* Big-SCAPE [22] was used to group the cluster into families and Cytoscape v3.6.1 was used to visualize the resultant network of cluster families.

## Additional files

Additional file 1: **Figure S1.** Network visualization of 35 gene cluster families in *Virgibacillus*, showing that few groups are found in the majority of *Virgibacillus* genomes and none are found in all nine genomes (DOCX 100 kb)

Additional file 2: **Table S1.** Features of modular clusters identified in *Virgibacillus* genomes (DOCX 16 kb)

Additional file 3: **Table S2.** Homology analysis of the modular genes in six clusters identified in Red Sea *Virgibacillus* strains to marine metagenomes. (DOCX 13 kb)

## Data Availability

All data used in this study have been included in this article.

## Abbreviations

A: Adenylation
AAMG: Automatic annotation of microbial genomes pipeline
ACP: Acyl carrier protein
AT: Acyltransferase
BGC: Biosynthetic gene clusters
C: Condensation
CAL: CoA ligase
E: Epimerization
GCF: Gene cluster family
GIs: Genomic islands
KR: Ketoreductase
KS: Ketosynthase
ML: Maximum likelihood
NRPS: Nonribosomal peptide synthetase
PCP: Peptidyl carrier protein
PKS: Polyketide synthase
RiPP: Ribosomally synthesized and post-translationally modified peptide
SMRT: Single-molecule real-time
T: Thioesterase
