# Supplementary material for: Mining biosynthetic gene clusters in Virgibacillus genomes
Source: BMC Genomics. 2019 Sep 3;20:696. doi: 10.1186/s12864-019-6065-7 (PMC6724285; doi:10.1186/s12864-019-6065-7)
Supplement: Supplementary file 1 — Figure S1. Network visualization of 35 gene cluster families in Virgibacillus, showing that few groups are found in the majority of Virgibacillus genomes and none are found in all nine genomes (DOCX 100 kb) [file 12864_2019_6065_MOESM1_ESM.docx]

**
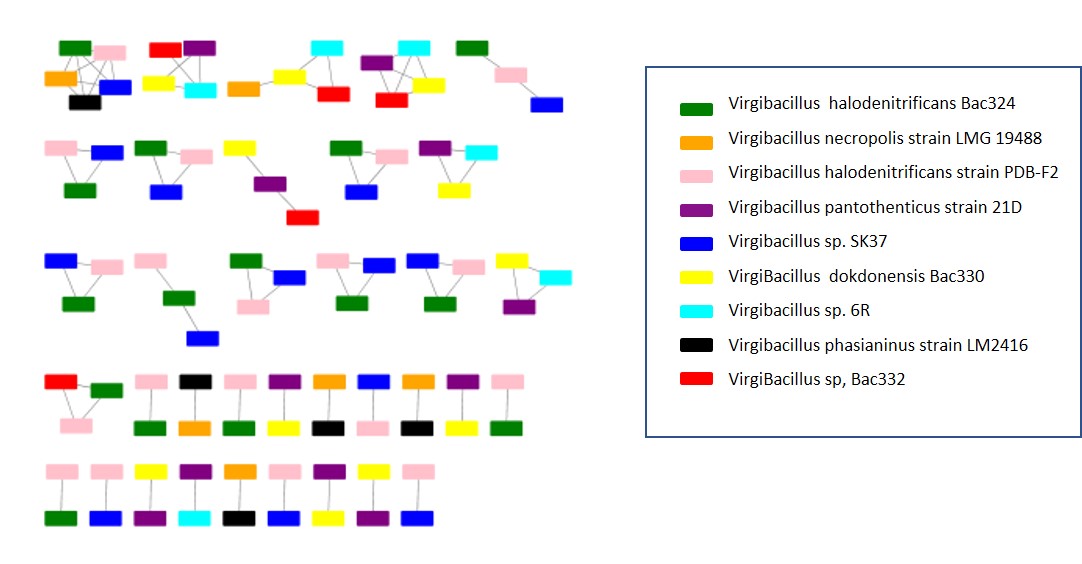
Figure S1**. Network visualization of 35 gene cluster families in *Virgibacillus*, showing that few groups are found in the majority of *Virgibacillus* genomes and none are found in all nine genomes.
