# Supplementary material for: Mining biosynthetic gene clusters in Virgibacillus genomes
Source: BMC Genomics. 2019 Sep 3;20:696. doi: 10.1186/s12864-019-6065-7 (PMC6724285; doi:10.1186/s12864-019-6065-7)
Supplement: Supplementary file 2 — Table S1. Features of modular clusters identified in Virgibacillus genomes (DOCX 16 kb) [file 12864_2019_6065_MOESM2_ESM.docx]

| **Genome** | **Accession No.** | **Cluster type** | **Start** | **End** | **Number of genes** | **Significant hits to known cluster** | **Complete and draft genomes with highest number of homologous genes** |
| --- | --- | --- | --- | --- | --- | --- | --- |
| Bac330 | CP033048 | hybrid Type III PKS/NRPS | 1710429 | 1801904 | 81 | 14% of genes show similarity to cereulide BGC | 97% similarity to genes in both *Virgibacillus dokdonensis* strain Marseille-P2545 and *Virgibacillus pantothenticus* strain 21D |
| Bac330 | CP033048 | NRPS | 2761865 | 2822901 | 38 | - | 69% similarity to genes in *Virgibacillus chiguensis* strain CGMCC 1.6496 |
| Bac332 | CP033046 | NRPS | 3330227 | 3381668 | 51 | - | 7 % similarity to genes in *Bacillus nakamurai* strain NRRL B-41091 |
| Bac332 | CP033046 | NRPS | 3878396 | 3928111 | 44 | 46% of genes show similarity to bacillibactin BGC | 100% similarity to genes in *Bacillus megaterium* MSP20.1 and 81% similarity to genes in *Virgibacillus massiliensis* strain Vm-5 |
| Bac332 | CP033046 | NRPS/ hybrid trans-AT PKS/NRPS | 2241455 | 2327734 | 43 | 64% of genes show similarity to locillomycin BGC | 86% similarity to genes in *Bacillus megaterium* MSP20.1 |
| BAC324 | Plasmid (CP033050) | NRPS | 124116 | 167403 | 42 | - | 7% similarity to genes in *Tumebacillus* sp. AR23208 |
| Sp. 6R | CP017762 | NRPS | 1073139 | 1133464 | 37 | 9% of genes show similarity to pacidamycin BGC | 25 % similarity to genes in *Marininema mesophilum* strain DSM 45610 |
| 21D | CP018622 | NRPS | 2572048 | 2633050 | 31 | - | 40% similarity to genes in *Virgibacillus dokdonensis* strain Marseille-P2545 |

**Table S1**. Features of modular clusters identified in *Virgibacillus* genomes
