# Supplementary material for: Mining biosynthetic gene clusters in Virgibacillus genomes
Source: BMC Genomics. 2019 Sep 3;20:696. doi: 10.1186/s12864-019-6065-7 (PMC6724285; doi:10.1186/s12864-019-6065-7)
Supplement: Supplementary file 3 — Table S2. Homology analysis of the modular genes in six clusters identified in Red Sea Virgibacillus strains to marine metagenomes. (DOCX 13 kb) [file 12864_2019_6065_MOESM3_ESM.docx]

**Table S2**. Homology analysis of the modular genes in six clusters identified in Red Sea *Virgibacillus* strains to marine metagenomes.

| **Genome** | **Cluster** | **# of modular genes in cluster** | **ID of metagenome with hit** | **Percent identities for modular genes** | **e-value for modular genes** |
| --- | --- | --- | --- | --- | --- |
| **Bac330** | hybrid Type III PKS/NRPS | 6 | MMP490065, MMP491463 | 49.612, 26.554, 31.505, 30.413, 30.017, 30.769 | 1.61e-137, 1.04e-09, 4.93e-164, 1.19e-127, 4.34e-81, 3.57e-34 |
| **Bac330** | NRPS | 7 | MMP492357, MMP490065, MMP492012, MMP491463 | 26.471, 24.627, 27.692, 37.778, 34.470, 26.304, 28.365 | 9.94e-18, 5.07e-86, 3.89e-145, 8.22e-48, 1.59e-75, 5.14e-78, 6.67e-19 |
| **Bac332** | NRPS | 2 | MMP490065 | 27.992, 48.684 | 0, 3.35e-48 |
| **Bac332** | NRPS | 4 | MMP494431, MMP490065 | 31.383, 31.760, 53.875, 45.313 | 2.14e-17, 0, 0, 7.67e-66 |
| **Bac332** | NRPS/ hybrid trans-AT PKS/NRPS | 8 | MMP491463, MMP494431, MMP490065 | 46.523, 38.190, 31.836, 31.910, 31.940, 32.439, 35.965, 34.868 | 1.26e-133, 3.56e-144, 0, 0, 0,0, 9.20e-48, 1.45e-21 |
| **BAC324** | NRPS | 2 | MMP494431, MMP490065 | 33.371, 27.536 | 2.02e-141, 8.34e-10 |
